# Factors Affecting Cirrus-HD OCT Optic Disc Scan Quality: A Review with Case Examples

**DOI:** 10.1155/2015/746150

**Published:** 2015-08-13

**Authors:** Joshua S. Hardin, Giovanni Taibbi, Seth C. Nelson, Diana Chao, Gianmarco Vizzeri

**Affiliations:** Department of Ophthalmology and Visual Sciences, The University of Texas Medical Branch at Galveston, Galveston, TX 77550, USA

## Abstract

Spectral-domain OCT is an established tool to assist clinicians in detecting glaucoma and monitor disease progression. The widespread use of this imaging modality is due, at least in part, to continuous hardware and software advancements. However, recent evidence indicates that OCT scan artifacts are frequently encountered in clinical practice. Poor image quality invariably challenges the interpretation of test results, with potential implications for the care of glaucoma patients. Therefore, adequate knowledge of various imaging artifacts is necessary. In this work, we describe several factors affecting Cirrus HD-OCT optic disc scan quality and their effects on measurement variability.

## 1. Introduction

The role of imaging in glaucoma management has significantly increased in recent years [1, 2]. Among the available imaging modalities, optical coherence tomography (OCT) has gained widespread acceptance, in part due to continuous technological improvements since its introduction in 1996 [3]. For example, modern spectral-domain (SD) OCT devices are characterized by enhanced resolution, reduced acquisition time and less operator dependence than time-domain (TD) OCT [4, 5]. Furthermore, hardware and software developments, including three-dimensional volumetric scanning protocols, have been incorporated in commercially available SD-OCTs, largely increasing the amount of information available to physicians. However, despite these remarkable advancements, recent evidence indicates that SD-OCT imaging artifacts are a common finding in clinical practice [6–9]. Poor scan quality can affect the ability of OCT to detect glaucoma and monitor its progression. Therefore, it is important for clinicians to identify the various OCT imaging artifacts and critically evaluate test results.

The purpose of the present work is to describe several common factors affecting Cirrus HD-OCT (Carl Zeiss Meditec, Dublin, CA; software version 5.0.0.326) scan quality and their effects on measurement variability using case examples collected in the clinic.

For narrative purposes only, we conveniently classified factors affecting Cirrus HD-OCT scan quality as patient-dependent, operator-dependent, and device-dependent. However, readers should be aware of the large overlap between these categories (i.e., scan artifacts often result from a combination of patient-dependent, operator-dependent, and device-dependent factors).

## 2. Patient-Dependent Factors

### 2.1. Pupil Size, Dry Eye, and Cataract

During scan acquisition, the OCT light beam is emitted toward the desired anatomic area, where the incident light is backscattered and then captured by the instrument for subsequent processing [3]. It follows that any obstacle to the pathway of the light beam may reduce the signal-to-noise ratio and interfere with the ability of Cirrus HD-OCT automated algorithm to identify the retinal nerve fiber layer (RNFL) boundaries or key optic nerve head features, such as the optic disc and cup margins, thus producing unreliable measures. Small pupil, for example, may potentially reduce the amount and quality of the signal detected by the instrument. However, Cirrus HD-OCT scans can be obtained in eyes with a pupil diameter greater than two millimeters. Recent studies did not find significant changes in RNFL thickness before and after dilation, suggesting that Cirrus HD-OCT results should not be affected by pupil size [10, 11]. However, pharmacological dilation may be necessary in select cases with small pupil size.

Conditions such as glaucoma, dry eye and cataract frequently coexist, due to their prevalence in the adult/aging populations [12–14]. In addition, ocular surface disease and dry eye syndrome are common in patients using topical ocular hypotensive drugs [15]. OCT studies have shown that dry eye and/or cataract diminish scan quality index and decrease RNFL thickness measures (see below) [16–21]. This effect should always be considered during cross-sectional or longitudinal evaluation of the RNFL, given the potential risk of false positives in glaucoma detection or disease progression.

**Figure 1:**
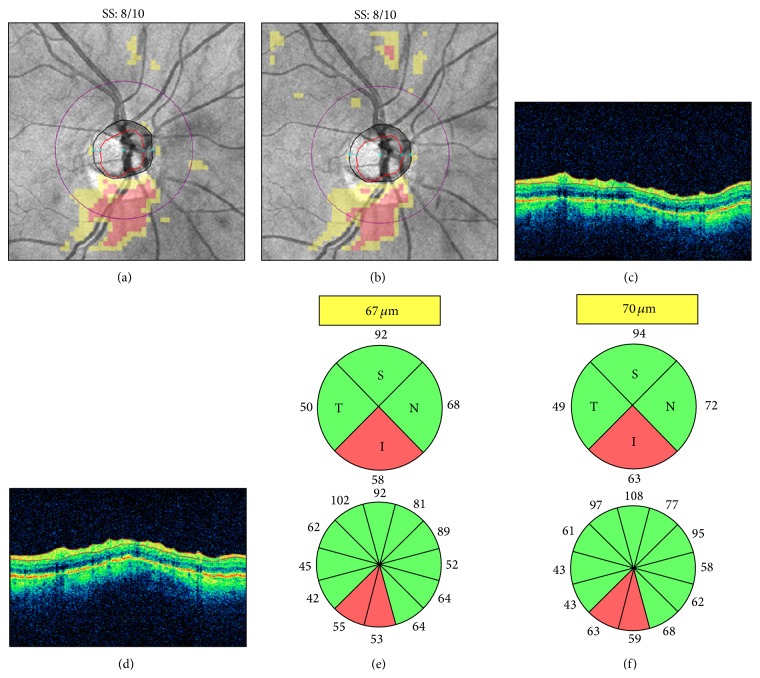
Dry eye. Cirrus HD-OCT retinal nerve fiber layer (RNFL) deviation maps ((a)-(b)), circular tomograms ((c)-(d)), and printout results ((e)-(f)) before (left) and after (right) instillation of artificial tears. Note the OCT color enhancement on the circular tomogram (d) and the overall increased RNFL thickness after adequate ocular lubrication (f). SS: signal strength.

Patients are commonly instructed not to blink during camera alignment and scan acquisition; however, this may cause tear film evaporation and breakup, particularly in patients with preexisting ocular surface disorders. Therefore, careful observation of the live funduscopic “en-face” image and the OCT tomograms is recommended, as it may reveal tear film disruption on the live image and signal degradation with color attenuation on the OCT tomograms. Patients should be encouraged to blink a few times immediately before scan capture to ensure uniform tear film distribution and to preserve adequate scan quality. This may also improve patient comfort, thus decreasing the likelihood of blinks or motion artifacts (see below) during scan acquisition. However, in some cases, artificial tears or gel may be necessary for adequate ocular surface lubrication (Figure 1).

Unfortunately, the detrimental effects of cataract on OCT scan quality are more difficult to overcome, unless cataract surgery is performed [17, 20]. Fine adjustments of the camera alignment may be attempted to purposely redirect the light beam through the areas of least cataract opacity to obtain acceptable image quality. Ultimately, cataract surgery should always be considered for optimal glaucoma management.

### 2.2. Floaters and Other Vitreous Opacities

**Figure 2:**
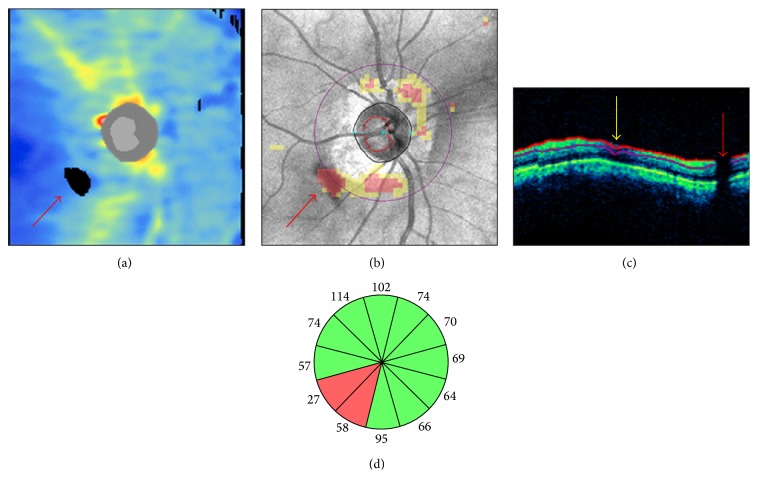
Floater overlying the scan circle. In a Cirrus HD-OCT right optic disc scan, a floater determined a black area of missing data on the retinal nerve fiber layer (RNFL) thickness map ((a) red arrow) and a cluster of red “superpixels” on the RNFL deviation map overlying the inferotemporal sectors of the scan circle (b). A characteristic vertical black shadow interrupted the retinal profile and the RNFL segmentation lines on the circular tomogram ((c) red arrow). A superonasal RNFL segmentation artifact from other cause is indicated by the yellow arrow. In (d), the inferotemporal clock hours underlying the floater presented RNFL thinning with “outside normal limits” classification results.

**Figure 3:**
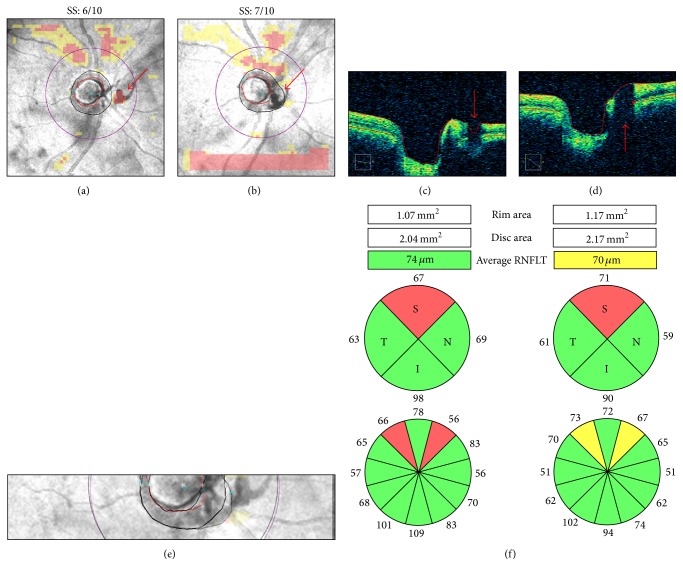
Floater overlying the optic disc region. Cirrus HD-OCT RNFL deviation maps ((a)-(b)), OCT tomograms ((c)-(d)), realigned en-face images (e), and printout results (f) from two right optic disc scans collected on the same day. In the first scan, the floater was located between the optic disc and the scan circle ((a) and (c), arrow). In the second scan, it was automatically included in the optic disc area ((b) and (d), arrow). The nasal margin of the floater was mistakenly interpreted as the optic nerve head neural canal opening ((d) asterisk), leading to increased rim and disc areas (f) and to inferonasal displacement of the optic disc center and the scan circle ((e) purple scan circle). Note the retinal nerve fiber layer thickening in the superotemporal clock hours, closer to the optic disc margin, and the corresponding thinning of the inferonasal clock hours, moved further away from the optic disc. SS: signal strength.

Floaters and other vitreous opacities have been documented in Cirrus HD-OCT macular and optic disc scans [22, 23]. These opacities may decrease scan quality by interfering with the light beam path, as described above. However, the effects on OCT measures are more closely related to their position within the scan area. When a floater is located on the scan circle (Figure 2(b)), a classical vertical shadow of signal attenuation/interruption is visible in the corresponding area of the circular tomogram (Figure 2(c)). Caution is recommended when interpreting the results, as the presence of the floater may mimic initial glaucomatous damage or falsely suggest thinning of a preexisting RNFL defect, particularly when it is located superotemporally or inferotemporally (Figure 2(d)). Although in most cases this artifact is easily identifiable on the printout, floaters near the optic disc area may remain undetected due to the presence of major retinal vessels and other graphical items displayed on the en-face image (Figure 3). Therefore, assessment of the tomograms intersecting the optic disc is warranted. In the example presented in Figure 3(b), a floater was mistakenly incorporated as part of the inferonasal optic disc area by the automated algorithm delineating the disc margins. Along with changes to optic disc parameters, the focal enlargement of the optic disc area dislocated the optic disc center and the scan circle inferonasally, producing major changes in RNFL thickness and classification results (Figures 3(e)-3(f)) [22].

Floaters may be successfully removed from the scan circle and the optic disc area by asking the examinee to produce brief to-and-fro eye movements immediately before scan acquisition.

### 2.3. Epiretinal Membranes

In addition to the floaters, other vitreopathies may be responsible for RNFL thickness changes. Recent studies have shown increased temporal and average RNFL thickness in eyes with epiretinal membrane (ERM), likely caused by ERM tractional forces on the retina [24, 25]. Therefore, caution is needed when interpreting RNFL thickness measures in eyes with ERM.

### 2.4. Blinks

Cirrus HD-OCT acquisition time is <2 seconds, making this test suitable for routine clinical use. However, blinks may still occur during this time frame. In the absence of an eye tracking system, the acquisition process continues uninterrupted even in the presence of blinks. This leads to a transient loss of data, which is proportional to the duration of the blink.

**Figure 4:**
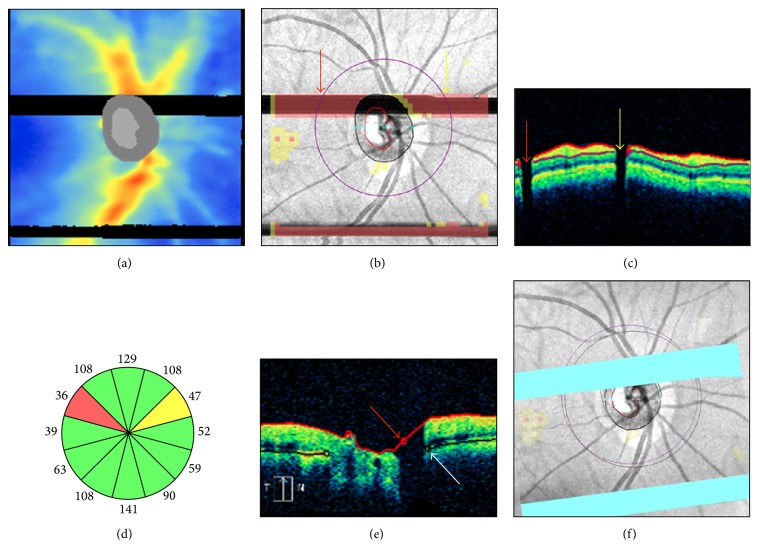
Blink artifacts. In a Cirrus-HD OCT right optic disc scan, two blinks produced two well-demarcated rectangular areas of missing data and red “superpixels” spanning the entire width of the retinal nerve fiber layer (RNFL) thickness map (a) and the RNFL deviation map (b). The upper blink affected the scan circle in two regions, as seen on the circular tomogram by two characteristic vertical black shadows interrupting the retinal profile (c). Note the corresponding RNFL thinning with abnormal classification results in clock hours 2 and 10 (d). The upper blink also affected the accuracy of the superior optic disc and cup margins ((e) arrows), leading to superotemporal scan circle displacement (f).

The effects of blinks on OCT measures depend on their position within the scan area. For example, Figure 4 shows two blink artifacts. The upper blink intersected the scan circle, causing RNFL thinning and abnormal classification results in the sectors affected by the artifact (Figure 4(d)). Also, the involvement of the superior pole of the optic disc led to inaccurate optic disc margin delineation and distortion of the optic disc shape, leading to superotemporal shift of the optic disc center with concomitant scan circle displacement (Figures 4(e)-4(f)).

In general, blink artifacts can be prevented by allowing the examinee to blink freely until completion of the camera alignment process, followed by prompt notification of the imminent start of scan acquisition. In selected cases, artificial tears or other lubricants may be indicated (see above).

### 2.5. Motion Artifacts

**Figure 5:**
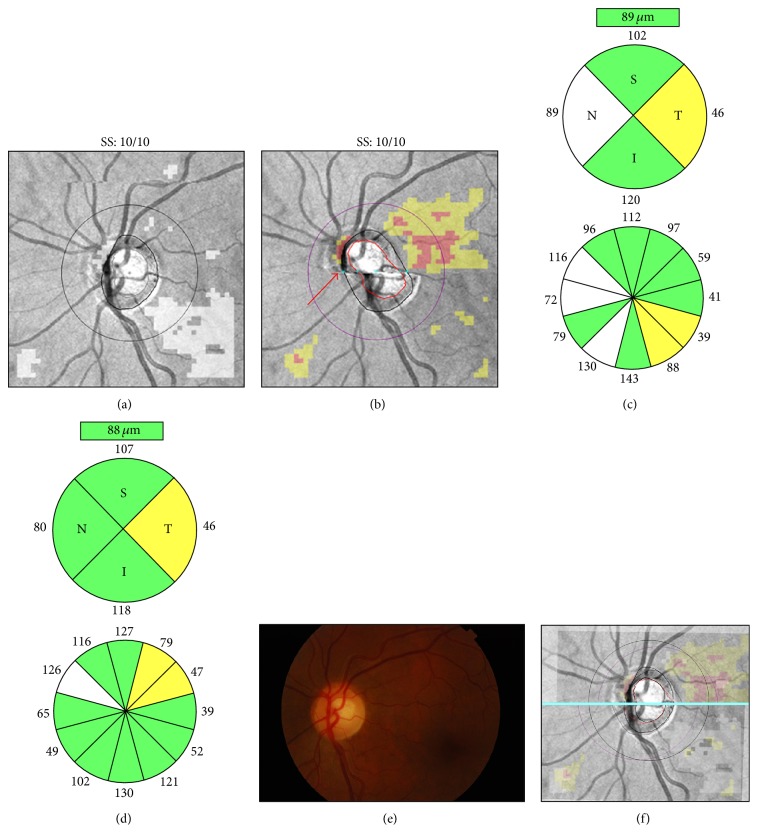
Motion artifacts and scan circle displacement. Pair of Cirrus HD-OCT left optic disc scans collected on the same day ((a)-(b)). In the scan with a motion artifact passing through the optic disc ((b) arrow), printout results suggest superotemporal retinal nerve fiber layer (RNFL) thinning not confirmed by color fundus photography ((d)-(e)). After realignment (f), this scan (purple scan circle) shows temporal displacement above the motion artifact and nasal displacement below the motion artifact. Note RNFL thickening in the sectors moved closer to the optic disc and RNFL thinning in the sectors moved away from the optic disc margin ((c)-(d)). SS: signal strength.

Motion artifacts result from eye movements, such as horizontal saccades, during scan acquisition. They typically appear on the en-face image as horizontal shifts of blood vessels' path (Figure 5(a)), but they may remain unnoticed when localized to the optic disc region or to areas without retinal vessels (Figure 5(b)). Improvements in SD-OCT scanning speed and acquisition time have reduced the likelihood of motion artifacts in OCT scans. However, eye movements still represent a potential problem for devices lacking an eye tracking system or motion correction algorithms. For example, Figure 5 shows a pair of Cirrus HD-OCT optic disc scans collected on the same day. The first scan is of adequate quality with a motion artifact away from the optic disc. The second scan, with a motion artifact passing through the optic disc, shows apparent superotemporal RNFL thinning not visible on color fundus photography (Figures 5(d)-5(e). This motion artifact induced optic disc shape distortion with shift of the optic disc center and scan circle displacement (Figure 5(f)). As a result, RNFL thickening was evident in the sectors closer to the optic disc margin, while RNFL thinning was seen in the sectors moved away from the optic disc, in agreement with previous studies [26–30]. Motion artifacts are a common finding in clinical practice and studies evaluating the effects of multiple motion artifacts on Cirrus HD-OCT measures are lacking.

Patient steady fixation is required to avoid motion artifacts. Therefore, clear explanation of the scanning procedures and timely notification of imminent image acquisition may be helpful. On-screen magnification of the en-face image may facilitate motion artifacts detection. For this purpose, the path of the retinal vessels and the optic disc shape should be carefully examined. Rescans should be attempted, particularly with motion artifacts intersecting the scan circle and/or the optic disc area. Careful interpretation of the results, including clock hours RNFL thickness, is warranted in scans with a motion artifact passing through the optic disc [30].

### 2.6. Signal Strength

The “strength” of the light signal backscattered by the ocular structures, calculated as signal to noise ratio, has been conventionally used as an objective measure of scan quality. Numerical scan quality scores are conveniently displayed on the printouts of commercially available SD-OCT instruments [31]. For example, Cirrus HD-OCT scan quality index, known as signal strength, ranges from 0 to 10. Only scans with signal strength ≥6 should be considered, as per manufacturer's recommendation. Therefore, signal strength maximization should be one of the operator's goals. However, as outlined above, several patient-related factors affect OCT scan quality and decrease the signal strength, including dry eye and media opacities [16–21]. In addition, operator-dependent factors, such as improper OCT lens cleaning or poor image centration, may also play a role (see below).

**Figure 6:**
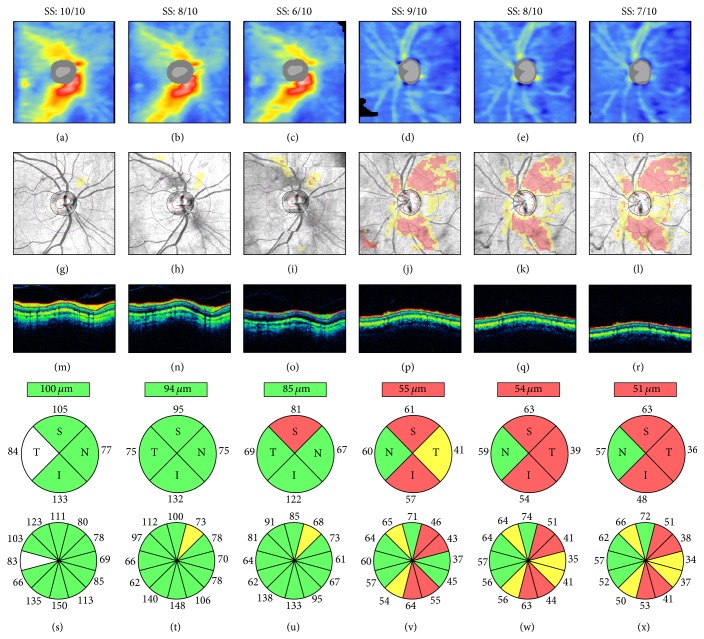
Signal strength and retinal nerve fiber layer (RNFL) thickness. Cirrus HD-OCT RNFL thickness maps ((a)–(f)), RNFL deviation maps ((g)–(l)), circular tomograms ((m)–(r)), and printout results ((s)–(x)) from two eyes. All scans had signal strength ≥6, the cut-off for acceptable image quality. Both cases show progressive signal attenuation ((m)–(o) and (p)–(r)), RNFL thinning and classification changes ((s)–(u) and (v)–(x)), and worsening of the RNFL thickness maps and RNFL deviation maps as signal strength decreases. Left: scans collected within a 2-week interval and sorted by decreasing signal strength; note the superotemporal reduction of the warmed-colored areas on the RNFL thickness maps, indicating RNFL thinning ((a)–(c)), and the corresponding clusters of “superpixels” on the RNFL deviation maps ((g)–(i)). Right: scans collected consecutively on the same day; note the progressive inferotemporal expansion of the “superpixels” cluster on the RNFL deviation maps ((j)–(l)). SS: signal strength.

Several independent studies have shown that scans with greater signal strength are associated with higher RNFL thickness measures [32–36]. This relationship also indicates that signal strength reduction is associated with decreased RNFL thickness, which may be erroneously interpreted as the presence of glaucomatous damage on a cross-sectional evaluation or as glaucomatous progression when multiple OCT scans are compared over time (Figure 6). Therefore, signal strength values should always be considered when evaluating RNFL thickness measures.

## 3. Operator-Dependent Factors

### 3.1. OCT Lens Opacities

**Figure 7:**
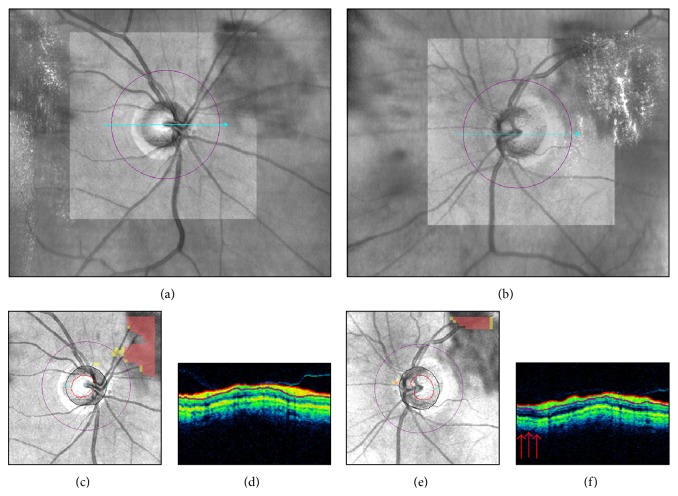
Smudged OCT lens. Live Cirrus HD-OCT funduscopic images ((a)-(b)), retinal nerve fiber layer (RNFL) deviation maps ((c), (e)), and circular tomograms ((d), (f)) from the two eyes of the same individual. A smudged OCT lens artifact in the superonasal and superotemporal peripapillary regions of the right and the left eyes, respectively, approached the corresponding sectors of the scan circle. In the right eye, note on the RNFL deviation map the yellow “superpixels” indicating localized RNFL thinning (c), despite apparent signal uniformity on the circular tomogram (d). In the left eye, note the discrete superotemporal area of signal attenuation on the circular tomogram ((f) arrows), possibly affecting the corresponding RNFL thickness measures.

Opacities of the OCT lens, such as those from fingerprints or accidental contact with the patient's periocular area or face (e.g., nose), may decrease image quality and directly affect RNFL thickness measures. In Figure 7, for example, smudged OCT lens artifacts approaching the scan circle are visible in the superonasal and superotemporal peripapillary areas of the right and left eyes of the same patient, respectively.

As shown in this example, OCT lens opacities typically maintain identical shape and occupy the same position on the en-face image over repeated testing. Periodic lens cleaning, as directed by the manufacturer, coupled with careful handling of the device by patients and test operators are necessary to prevent the occurrence of these artifacts.

### 3.2. Incorrect Axial Alignment of the OCT Image

**Figure 8:**
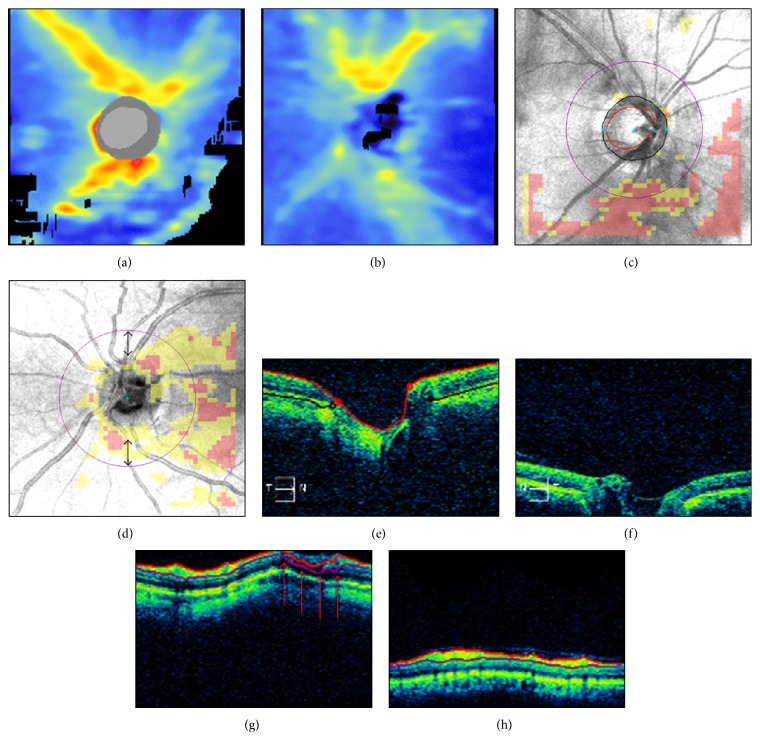
Incorrect axial alignment. Cirrus HD-OCT retinal nerve fiber layer (RNFL) thickness maps ((a)-(b)), RNFL deviation maps ((c)-(d)), horizontal ((e)-(f)), and circular ((g)-(h)) tomograms from two eyes. Left panels: partial truncation of the inner retinal layers; note the black areas of missing data on the RNFL thickness map (a), the corresponding clusters of “superpixels” on the RNFL deviation map (c), and the retinal nerve fiber layer segmentation artifact ((g) arrows). Right panels: partial truncation of the optic disc cup (f); note the algorithm failure to detect the optic disc (see (b), (d), and (f) and compare with the corresponding left panels). Thus, the scan circle was automatically placed in the center of the image, resulting in inferior scan circle displacement ((d) double arrows).

Improper axial alignment occurs when the ocular structures are only partially included within the acquisition frame, resulting in image truncation. In Cirrus HD-OCT scans, the innermost (e.g., peripapillary inner retinal layers) and outermost features (e.g., optic disc cup) are particularly vulnerable to this type of artifact, due to their proximity to the anterior and posterior edges of the acquisition window, respectively. Two examples are presented in Figure 8. In the first case, the inferonasal and inferotemporal peripapillary areas show truncation of the inner retinal layers extending to the inferior quadrant of the scan circle, thus producing inaccurate RNFL delineation (Figure 8(g)). In the second case, there is partial truncation of the optic disc cup, leading to algorithm failure to localize the optic disc features (Figure 8(d)).

OCT image truncation may commonly occur in myopic eyes with steep retinal curvature or in glaucomatous eyes with deep optic disc cupping. Other causes include improper distance between the eye and the device due to incorrect patient positioning, or axial misalignment of the OCT scanning head. Along with continuous monitoring of the axial alignment during the imaging procedures, Cirrus HD-OCT users are encouraged to use the “optimize” feature for automated scan alignment and quality optimization. Proper patient positioning is also important in preventing head tilt and subsequent image rotation, another known cause of RNFL thickness measurement variability [37].

## 4. Device-Dependent Factors

### 4.1. Inaccurate Optic Disc Margins Delineation

**Figure 9:**
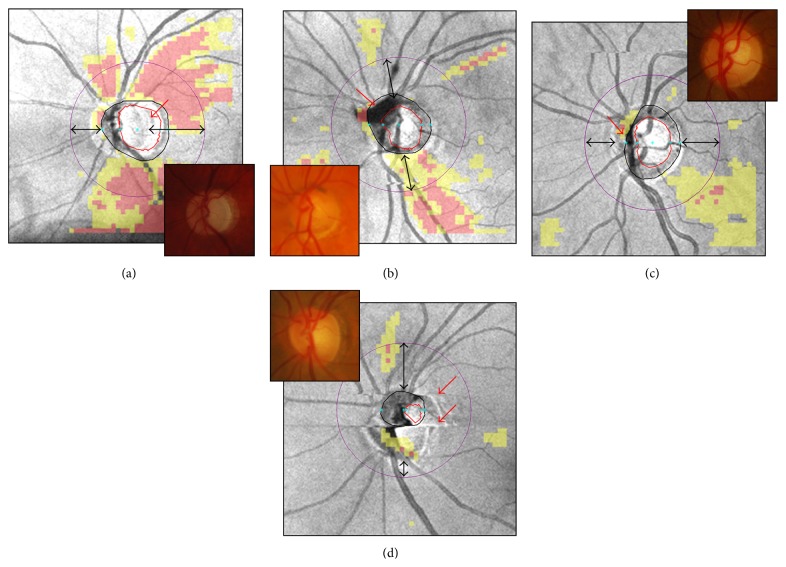
Cirrus HD-OCT inaccurate optic disc margin delineation from peripapillary atrophy, floater, blood vessels' pattern, and motion artifacts ((a)–(d), red arrows). Color optic disc photographs are shown in the insets for comparison. Along with unreliable optic disc parameters (not shown), note the scan circle displacement in all cases ((a)–(d), double arrows).

Cirrus HD-OCT provides a series of optic disc parameters (e.g., optic disc area, rim area, average and vertical cup-to-disc ratios, and cup volume) through automated delineation of the optic disc and cup margins. Adequate optic disc assessment relies on the ability of the automated algorithm to identify the termination of the Bruch's membrane, corresponding to the optic disc edge [38, 39]. In addition, accurate delineation of the optic disc area is necessary to calculate the optic disc center and place the scan circle evenly around it. For example, Figure 9 illustrates two examples of optic disc area overestimation in scans with peripapillary atrophy (PPA) and a floater near the optic disc and two examples of optic disc area underestimation from interference by blood vessels and motion artifacts, all causing scan circle displacement. In the presence of PPA, alterations of the OCT signal reflectance from retinal pigment epithelium disruption and choriocapillaris atrophy [40], coupled with ultrastructural Bruch's membrane changes [41], possibly affected the correct identification of the optic disc margin (Figure 9(a)). The vertical shadows of signal interruption induced by the floater and the particular blood vessels' pattern in the optic disc region were mistakenly interpreted as the optic nerve head neural canal opening (Figures 9(b)-9(c)). Finally, the severe optic disc shape distortion induced by two motion artifacts determined obvious misidentification of the optic disc structures (Figure 9(d)). As seen above, blinks or optic disc cup truncation may also lead to unreliable or absent optic disc parameters.

In each scan, careful inspection of the en-face image and the tomograms intersecting the optic disc is necessary to evaluate the accuracy of the optic disc outlining process. Because manual correction of the disc margins is not allowed, rescans should be attempted to obtain accurate optic disc margin delineation.

### 4.2. Inaccurate Retinal Nerve Fiber Layer Segmentation

**Figure 10:**
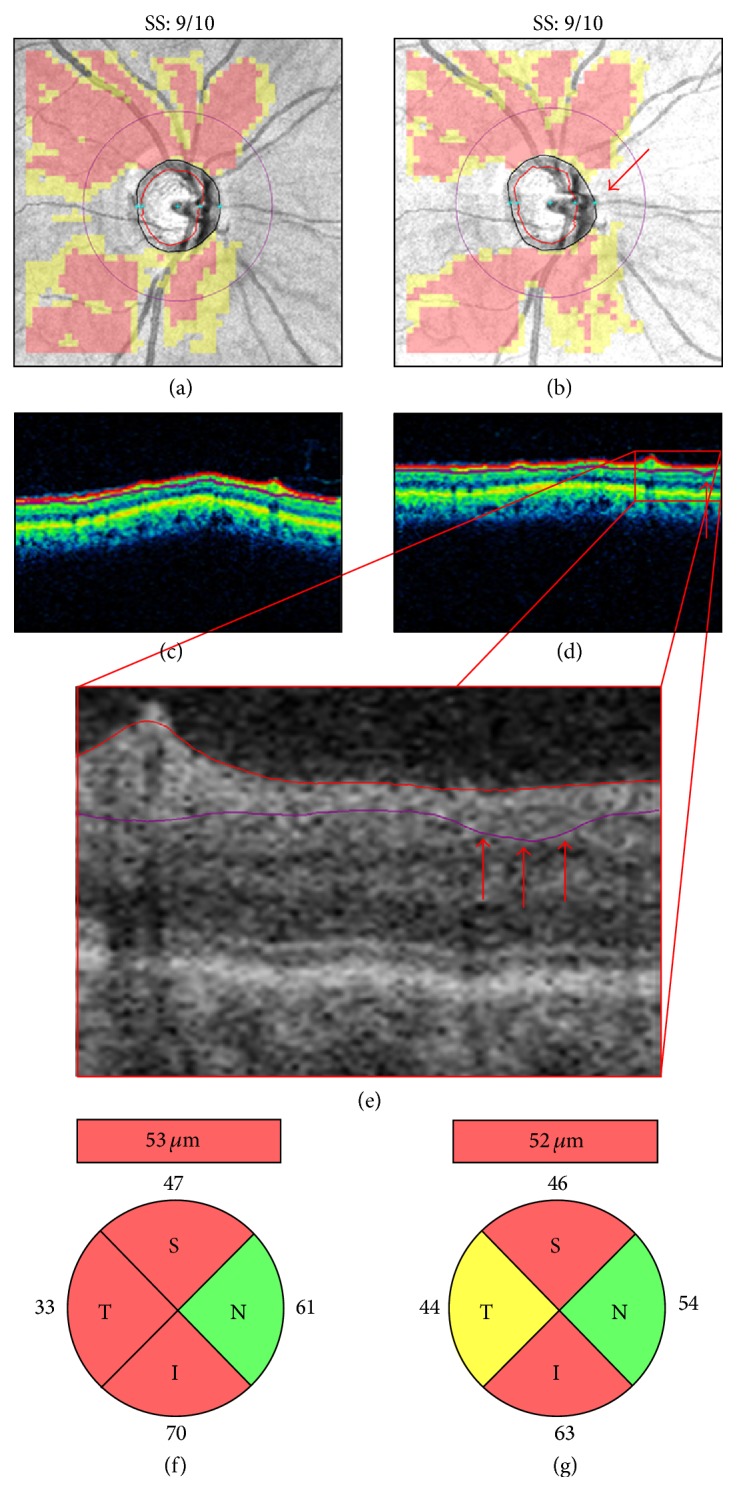
Inaccurate retinal nerve fiber layer (RNFL) segmentation. Pair of right optic disc scans collected on the same day ((a)-(b)). The second scan presents with a motion artifact intersecting the temporal and nasal quadrants of the scan circle ((b) arrow), which is likely responsible for the subtle RNFL segmentation error ((d)-(e), arrows) and the resulting temporal RNFL thickening with classification change ((f)-(g)). SS: signal strength.

Estimation of the RNFL thickness relies on the ability of Cirrus HD-OCT to distinguish the RNFL from the other retinal layers, a process known as segmentation. Several mechanisms may be responsible for inaccurate RNFL segmentation, such as OCT signal attenuation with decreased reflectance of the RNFL induced by ocular media opacities (see also Figure 2(c), yellow arrow). The OCT signal may even be partially interrupted as per the effect of blinks or floaters (Figures 2 and 4), causing localized failure to identify the RNFL boundaries and decreased RNFL thickness measures. Furthermore, truncation of the inner retinal layers may determine algorithm failure or obvious RNFL segmentation errors (Figure 8(g)). Finally, motion artifacts intersecting the scan circle may facilitate inaccurate RNFL segmentation (Figure 10).

After scan acquisition, careful assessment of the circular tomogram is necessary to exclude RNFL segmentation artifacts. On-screen magnification of the circular tomogram is advisable. In addition, the grayscale view may show fine retinal details and segmentation errors that could be easily missed using the standard false-color visualization mode (Figure 10(e)) [3].

## 5. Conclusions

This paper described factors affecting Cirrus HD-OCT optic disc scan quality and their effects on measurement variability. As a reference guide for imaging technicians and interpreting physicians, these factors are summarized in Table 1.

Although Cirrus HD-OCT is remarkably less operator-dependent than the previous TD-OCT, it appears that OCT operators (e.g., technicians) still play a pivotal role. First, they should be familiar with all known imaging artifacts and adopt the necessary provisions to prevent or minimize their occurrence. Second, they are typically the first professionals performing a scan quality check, an essential task that should always precede the interpretation of test results. For this purpose, careful examination of the different graphical and numerical items of the output is warranted, as demonstrated in the above examples. Moreover, on-screen image magnification may prove extremely useful in detecting imaging artifacts otherwise not easily identifiable on the printout, such as motion artifacts intersecting the optic disc or RNFL segmentation errors. Prompt recognition of imaging artifacts makes rescan attempts feasible and time-efficient, as rescans are most easily obtained when the patient is still seated at the instrument.

The examples presented also indicate that scan circle displacement remains possible with Cirrus HD-OCT, despite built-in automated algorithm for scan circle positioning around the optic disc. Scan circle displacement should be suspected in case of blinks, floaters, or motion artifacts involving the optic disc area, and careful inspection of the optic disc contours is recommended for each scan.

It should be emphasized that SD-OCT devices have different technical specifications, scanning protocols and detection algorithms [42, 43]. Therefore, not all factors affecting measurement variability discussed in this paper using Cirrus HD-OCT case examples may apply to other SD-OCT instruments. For example, results from Spectralis OCT (Heidelberg Engineering, GmbH, Heidelberg, Germany), the new generation of RTVue OCTs (Optovue Inc., Fremont, CA), or the latest Cirrus HD-OCT 5000 (see Hwang and associates [44]), with their built-in eye tracking systems, should be less affected by blinks or motion artifacts. On the other hand, it is conceivable that dry eye and cataract, floaters, changes in scan quality index, OCT lens opacities, or incorrect axial alignment may exert similar effects among different SD-OCT devices.

The present work focused on Cirrus HD-OCT imaging artifacts and did not cover other important sources of measurement variability specifically related to the patients' anatomical features, such as axial length and refraction [45–51]. For example, the Littman formula can be used to correct axial length-related ocular magnification errors, which typically occur in myopic eyes: *t* = *p* · *q* · *s*, where *t* is the actual size of the ocular structure, *p* is the imaging system magnification factor (i.e., *p* = 3.382 for Cirrus HD-OCT), *q* is the ocular magnification factor (*q* = 0.01306 · [axial length − 1.82]), and *s* is the measurement as provided by the instrument [52].

In conclusion, continuous software and hardware implementations make SD-OCT a dynamic and evolving imaging modality. However, clinicians should be aware of its limitations, and adequate knowledge of possible scan artifacts is necessary for critical interpretation of the results. Clinical decisions should never be driven by ocular imaging tests only. Rather, a thorough ophthalmological examination and complete visual function assessment remain essential tools in the management of glaucoma patients.

## Figures and Tables

**Table 1:** Factors affecting Cirrus HD-OCT scan quality.

	Patient-dependent					Operator-dependent		Device-dependent	
	Dry eye and cataract	Floaters/vitreous opacities	Blinks	Motion artifacts	Signal strength	OCT lens opacities	Incorrect axial alignment	Inaccurate optic disc margin delineation	Inaccurate RNFL segmentation
Visible as		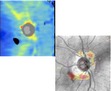	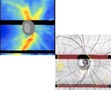	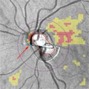	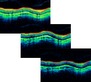	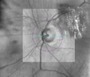	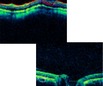	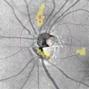	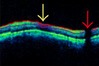
	Tomograms: signal attenuation	(i) RNFLT map: cold-colored/black area of missing data (ii) RNFL deviation map: clusters of “superpixels” (iii) Tomograms: vertical shadow of signal interruption	(i) RNFLT and deviation maps: well-defined rectangular black area of missing data, red “superpixels” (ii) Tomograms: absence of signal; or vertical shadow of signal interruption	En-face image: horizontal shift of the retinal blood vessels' path; optic disc shape distortion	Numerical scan quality index, displayed on the printout (range: 0–10)	(i) RNFLT map: cold-colored/black area of missing data (ii) RNFL deviation map: clusters of “superpixels” (iii) Tomograms: signal attenuation	(i) RNFLT map: crescent-shaped black area of missing data (ii) RNFL deviation map: clusters of “superpixels” (iii) Tomograms: image truncation	En-face image: malposition of the optic disc and/or cup contour lines	Circular tomogram: malposition or interruption of RNFL segmentation lines

Effects	(i) ↓ Signal strength (ii) ↓ RNFLT	(i) Floater on scan circle: ↓ RNFLT (ii) Floater on optic disc area: unreliable optic disc parameters, possible scan circle displacement with RNFLT measurement variability	(i) Blink affecting optic disc: unreliable optic disc parameters, possible scan circle displacement with RNFLT measurement variability (ii) Blink affecting scan circle: ↓ RNFLT	(i) Inaccurate optic disc margin delineation, unreliable optic disc parameters (ii) Scan circle displacement with RNFLT measurement variability	Low signal strength associated with ↓ RNFLT	↓ RNFLT	(i) Inner retina truncation: inaccurate RNFL segmentation (ii) Optic disc truncation: unreliable optic disc parameters, possible scan circle displacement with RNFLT measurement variability	(i) Unreliable optic disc parameters (e.g., ↑ or ↓ disc area, depending on the cause) (ii) Possible scan circle displacement with RNFLT measurement variability	↑ or ↓ RNFLT, depending on the cause

Suggestions	(i) Blinks prior to scan acquisition (ii) Lubricants for dry eyes (iii) Redirect light beam through areas of least cataract opacity	Ask patient to perform brief to-and-fro eye movements immediately before scan acquisition to displace floater	(i) Blinks prior to scan acquisition (ii) Lubricants for dry eyes (iii) Prompt notification of imminent scan acquisition	(i) Blinks prior to scan acquisition (ii) Lubricants for dry eyes (iii) Prompt notification of imminent scan acquisition (iv) Verify: continuity of blood vessels' path, optic disc shape, accuracy of optic disc margin delineation	Identify and address cause (e.g., OCT lens cleaning, adjust camera alignment, lubricants, pupil dilation)	(i) Careful instrument handling (ii) Periodic OCT lens cleaning	(i) Proper patient positioning (ii) “Optimize” feature for automated axial alignment	Identify and address cause (e.g., floaters, motion artifacts)	Identify and address cause (see patient- and operator-dependent factors)

RNFLT: retinal nerve fiber layer thickness.

Note: case examples obtained using Cirrus HD-OCT (Carl Zeiss Meditec, Dublin, CA; software version 5.0.0.326). The content of this table may not be applicable to different Cirrus HD-OCT models or to other Spectral-domain OCT devices.
